# SUPPORTING LATE AND POST–CAREER STAGE FACULTY: AGE-INCLUSIVE OPPORTUNITIES FOR PROFESSIONAL ORGANIZATIONS

**DOI:** 10.1093/geroni/igae098.0990

**Published:** 2024-12-31

**Authors:** Joann Montepare, Rowena Gomez, Harvey Sterns

**Affiliations:** Lasell University, Newton, Massachusetts, United States; Palo Alto University, Palo Alto, California, United States; University of Akron, Akron, Ohio, United States

## Abstract

Our populations are aging nationally and locally at dramatic rates as we experience extended longevity, with significant implications on many fronts. With higher education employing a greater proportion of individuals over the age of 65 relative to the general labor force, more faculty will be moving into their late and post career stages at their institutions in the coming years. Thus, campuses are natural environments where long-time faculty members are “aging in the workplace.” For organizations like the American Psychological Association (APA) and GSA, many of its members are affiliated with higher education and are part of this demographic trend. Aiming to inform our professional organizations about how they can better support these (and other) members, this presentation will describe the results of a survey of 391 APA members (age 52+) who responded to questions about how the association could support members’ professional development, retirement experiences, and opportunities for further engagement and support. A variety of recommendations emerged that can be translated easily to opportunities for GSA (e.g., develop dialogue and related programming around members’ career transitions and retirement planning that speak to financial challenges and physical or medical concerns, as well as sustaining professional contributions and maintaining social and emotional connections with colleagues at similar career stages). We as scholars in the aging field know well the value of a lifespan development lens, which we now need to use in a more self-reflective way in connection with our professional organizations.

